# An emotion analysis in learning environment based on theme-specified drawing by convolutional neural network

**DOI:** 10.3389/fpubh.2022.958870

**Published:** 2022-11-03

**Authors:** Tiancheng He, Chao Li, Jiayang Wang, Minjun Wang, Zhenghao Wang, Changyong Jiao

**Affiliations:** ^1^School of Social Science, Zhejiang University of Technology, Hangzhou, China; ^2^Department of Political Party and State Governance, East China University of Political Science and Law, Shanghai, China; ^3^Zhijiang College, Zhejiang University of Technology, Shaoxing, China; ^4^College of Materials Science and Engineering, Zhejiang University of Technology, Hangzhou, China; ^5^School of Economics, Hefei University of Technology, Hefei, China; ^6^School of Marxism, Lanzhou University, Lanzhou, China; ^7^School of Management, Zhejiang University of Technology, Hangzhou, China

**Keywords:** convolutional neural network, intelligence healthcare, machine learning, emotional analysis, learning environments

## Abstract

Emotion in the learning process can directly influence the learner's attention, memory, and cognitive activities. Several literatures indicate that hand-drawn painting could reflect the learner's emotional status. But, such an evaluation of emotional status, manually conducted by the psychologist, is usually subjective and inefficient for clinical practice. To address the issues of subjectivity and inefficiency in the painting based emotional analysis, we conducted an exploration of a painting based emotional analysis in learning environment by using convolutional neural network model. A painting image of 100 × 100 pixels was used as input for the model. The instant emotional statue of the learner was collected by filling out a questionnaire and was reviewed by a psychologist and then used as the label for training the convolutional neural network model. With the completion of convolutional, full-connected, and classification operations, the features of the painting image were learned from the underlying pixel matrix to the high-level semantic feature mapping. Then the emotional classification of the painting image could be made to reflect the learner's emotional status. Finally, the classification result by the model was compared with the result manually conducted by a psychologist to validate the model accuracy. We conducted an experiment in a university at Hangzhou, and 2,103 learners joined in the experiment. The learner was required to first fill out a questionnaire reporting emotional status in the learning process, and then to complete a theme-specified painting. Two thousand valid paintings were received and divided into training dataset (1,600) and test dataset (400). The experimental result indicated that the model achieved the accuracy of 72.1%, which confirmed the effectiveness of the model for emotional analysis.

## Introduction

Emotion is a mental response caused by external stimuli that influences and regulates cognitive activities (such as perception). Siemens (1) argued that emotion is the “gatekeeper” of our neural networks, and it is important to combine cognition and emotion in a learner's understanding. Literatures indicate that positive emotions such as concentration, happiness and satisfaction during the learning process help to stimulate motivation, develop interest in learning and promote cognitive activity, meanwhile negative emotions such as sadness and anxiety affect concentration, patience and hinder cognitive activity (2, 3). In learning environments such as MOOCs, teachers and learners find it hard to feel each other's emotional states due to the quasi-spatial and temporal separation, hence there is a general problem of “emotion deficit” (4). Researchers have less consideration for the roles of non-intellectual factors such as emotion and motivation in the learning process. As a result, the study of learners' psychological emotions in learning environments received increasing attention with regards to various kinds of fields (such as psychology) (5–7). A well-known test in modern psychology, which has been proved as scientifically valid in measurement efficacy, is the tree drawing (8). It can be used as an indicator of the subject's emotional state. However, this type of test is usually carried out under the supervision of a professional psychologist, and that would involve manually handling a huge amount of painting images. Such a manual analysis process is subjective and ineffective.

A lot of researchers introduced machine learning to address the issues of subjectivity and inefficiency in the image sentiment analysis (8–10). Emotional analysis of hand-drawn painting refers to the detection and recognition of information related to emotions hidden in the painting image (1, 2), which is the analysis of the emotional information expressed and conveyed by the image from a perspective other than that of the image. If the image is considered as an information carrier, the image would be used as a tool by its creator to express personal emotions. Traditional machine learning methods (e.g., support vector machine, linear classifier, back propagation neural network) first extract the low-level visual features of the image (e.g., color and its distribution, texture and lines, shape, and its spatial layout), then train the emotion classifier using training samples, and finally use the trained classifier to identify the emotions of the image (11). However, these traditional machine learning methods are not suitable for this context because the hand-drawn paintings usually contain highly abstracted content, more space, and less details.

Convolutional Neural Network (CNN) has been widely adopted in areas such as image processing and natural language processing, but is rarely mentioned in the field of emotional analysis (3). CNN combines image feature extraction with fuzzy classification by neural networks, omits the complex image pre-processing and feature extraction process, makes it no longer dependent on manually crafted explicit feature extraction, and improves efficiency and accuracy.

In the paper, we proposed a CNN-based model to carry out the emotional analysis of learners in the learning process. The painting images completed by the learners are used as the input of the model. The emotional statues reported by the learners and reviewed by the psychologists are used as the labels for training the convolutional neural network model. With the trained model, the emotional classification of the painting images could be efficiently achieved to reflect the learners' emotion statues. The contribution in this work is summarized as:

1) A novel method to model and analyse the emotional statues of the learners in a learning environment, in which CNN is incorporated to accelerate the analysis process and improve the classification accuracy.
2) A 6-layer neural network structure to extract the semantic and sequential features of the hand-drawn paintings (e.g., highly abstracted content), is proposed. The structure supports the use of image pixel values directly as input to implicitly obtain the abstract feature information of a painting image.
3) A hybrid method for data labeling is proposed, in which, a designated theme (i.e., tree) hand-drawn painting task is introduced as a tool to collect dataset, the self-reported emotional statues (by filling a questionnaire form), which are reviewed (revised if needed) by the psychologists, will be used as labels for training the emotion classifier. And an experiment including 2,103 participants has confirmed the effectiveness of the method.

The article is organized as below. The related literature is reviewed in the Section Related work. The detail of the proposed method are presented in Section Method. The experiment is introduced in Section Experiment and discussion. The Section Conclusion concludes the work.

## Related work

### Emotion analysis

Charles Darwin pointed out that emotions were adaptive responses to external stimuli developed by individuals over the course of evolution, and that there are many basic emotions (1). Descartes argued that humans have six primitive emotions such as surprise, pleasure, hatred, desire, and sadness, and that all other emotions are combinations of them (2). Based on the study of facial expressions and behavioral responses, Kang (6) classified the basic emotions into six, such as delight, surprise, sadness, anger, fear, and disgust. Izard (3) proposed a theory of differential emotions, arguing that basic emotions include shyness, contempt, interest, and self-guilt. Plutchik (12) argued that emotions have three dimensions (i.e., intensity, bipolarity, and similarity), and accordingly proposed eight basic emotions (i.e., grief, fear, surprise, acceptance, ecstasy, rage, vigilance, and hatred). In summary, the main categories of emotion models include: (1) those based on basic emotion theory; (2) those based on cognitive mechanisms and (3) those based on personalization. The Orton Clore Collins (OCC) model was proposed by Orton, Clore and Collins in 1988 and has been widely used in the design of emotion models (13). The OCC classifies emotions into three categories based on their causes: (1) the outcome of an event, (2) the actions of an intelligent agent, and (3) the perception of an object.

Emotion is essential for perception, reasoning, decision-making and creativity, and is already an important part of intelligence. Similarly, emotion plays a key role in social interaction, and Picard (14) introduced the concept of Affective Computing that involves, originates from, or intentionally affects aspects of emotion. In simple terms, this means that computers are expected to have human-like abilities to observe, understand and generate a variety of emotions. The fundamental goal of affective computing is to attempt to create an “intelligent” computing system that can sense, recognize and understand human emotions, and provide intelligent, timely and friendly feedback based on human emotions. In 1995, Picard introduced the Hidden Markov Model (HMM) to affective computing, which has three affective states, interest, happiness, and sadness, but can be expanded to more than one (15). E.g., a fourth ellipse could be added to represent the “no emotion state,” as an emotional baseline or neutral state. Picard argues that a person's emotional state cannot be directly observed but can be judged by observing external features. Thus, it is possible to use features to determine their possible corresponding affective states, or to use the states described by the entire HMM structure map to identify larger scale affective behaviors. The latter would undoubtedly require a larger number of HMM structure maps, each corresponding to one affective behavior, and HMMs can represent either a mixture of several affects or a mixture of several pure affective states that emerge in a constant alternation based on time (4, 16).

### Artificial neural networks

Artificial Neural Networks (ANNs) have gone through three phases to date, the first of which began in cybernetics in the 1940s and 1960s. However, it was unable to handle “heterogeneous” problems and computers did not have sufficient computing power to run neural networks for a long time. The second phase began in the late 1980s with the Back Propagation (BP) algorithm by Rumelhart (11), which overcame the “heteroskedasticity” problem and reduced the amount of complex computation required for two-layer neural networks. In 2006, Hinton et al. proposed a neural network model called Deep Belief Network (DBN) to achieve data dimensionality reduction (17). Its core ideas include: (1) neural network structures with more hidden layers have unique feature learning capabilities and can better capture the essential features of images; (2) the training difficulty of deep neural networks can be overcome by “layer-by-layer” initialization.

Deep learning is a generic term for a class of methods that train models with deep structure. The mainstream deep learning models include Deep Belief Networks (DBN), Recurrent Neural Networks (RNN) and Convolutional Neural Networks (CNN). In 1998, Yann proposed a gradient learning-based CNN algorithm and applied it to handwritten numeric character recognition (18). CNN was originally inspired by neuroscience and the visual information processing of simple and complex cells in the visual nerve. They usually use convolution operations to simulate the processing of edge information in different directions by simple cells and pooling operations to simulate the cumulative processing of similar simple cells by complex cells. The CNN supports the use of image pixel values directly as input to implicitly obtain the abstract feature information of an image without pre-processing of the image and explicit extraction of image features, avoiding the complex feature extraction and manual selection process. CNN is highly robust for transformations (e.g., translation, scaling) and rotation of images as well as sensitive problems (e.g., illumination and occlusion) (19). Hand-drawn image classification using Fisher Vector proposed by Schneider et al. (20) achieved better recognition features, and its recognition accuracy can be close to that of humans. In 2015, Yu et al. (5) proposed the Sketch-a-Net, which is especially designed to address hand-drawn image recognition. It adopts the fusion of multi-scale networks by means of Bayesian fusion, which can effectively solve the extraction and sparsity problems of hand-drawn sketches.

Quaglia et al. (8) achieved the recognition of image emotion based on color and texture. Badrulhisham et al. (9) fused visual features such as color, texture and shape and used Support Vector Machine (SVM) to achieve the mapping of comprehensive image feature space to emotion space. Farrokhi et al. (10) pointed out that the role of visual features such as shape, color and texture of images in perception would lead to different psychological responses. Riedl (21) proposed that different color combinations produce different image emotions. By combing through the literature related to image emotion classification (7, 22), pointed out that the existing image emotion analysis is mostly based on the study of low-level visual features such as color, shape and texture of images. Therefore, from the generation principles and established studies, learning image emotion is usually influenced by low-level visual features such as color, texture, and shape.

## Method

In this study, we use a hand-drawn painting-based method to classify the emotional statue of the learner, and each painting contains multiple labels, hence a multi-label classification model is required. Based on the analysis previously, we took CNN to achieve such a target. A typical CNN structure usually includes an input layer, convolutional layer, pooling layer, fully connected layer, and output layer. A painting image in this context usually contains complex low-level visual features such as color, texture and shape. There is a “semantic gap” between the low-level visual features and the high-level emotional semantics. Therefore, a 6-layer CNN structure is adopted for emotion classification of hand-drawn painting images in this context.

### The model structure

The hand-drawn image is more complex than the computer-generated image in terms of lower-level visual features (e.g., color, texture and shape), so the size of the input image is set to 100 × 100 pixels to allow the CNN to extract more low-level visual features. Although the use of small convolutional kernels can increase the depth of the network and reduce the number of parameters, the size of the input image is 100 × 100 pixels, which is large. To significantly enlarge the receptive field, the size of the first convolutional kernel is set to 7 × 7, while the size of the other convolutional kernels is set to 5 × 5, and the convolutional step size is set to 1.

**Figure 1:**
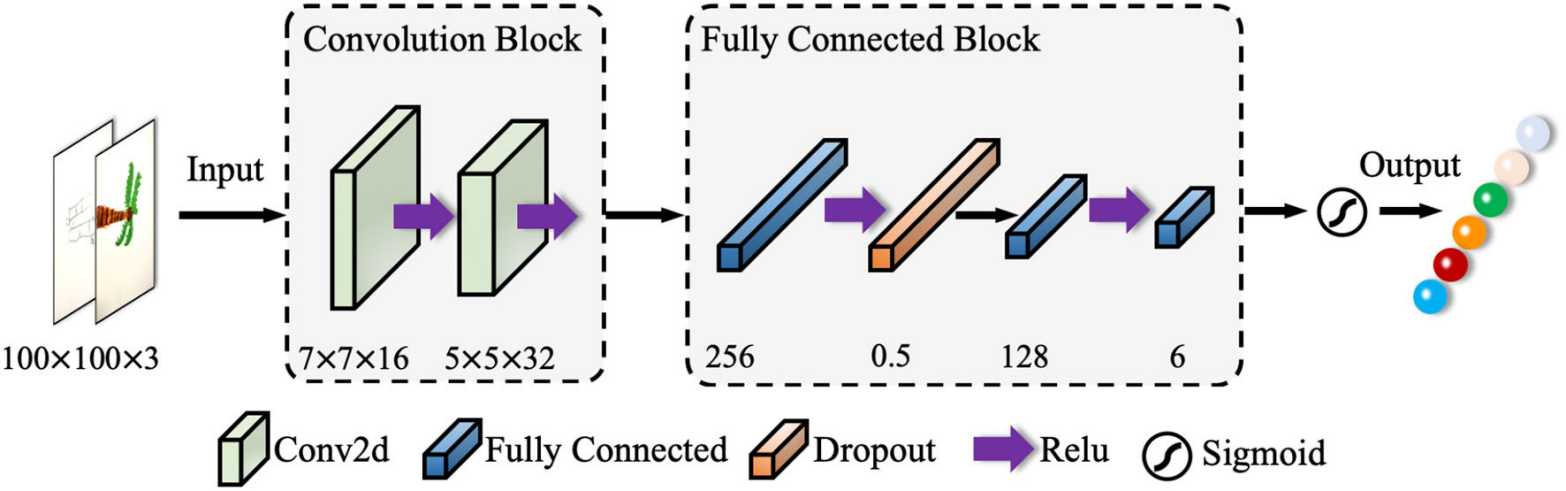
Structure of the network adopted in this context.

The pooling layer is disabled in this structure to reduce feature loss. The activation function (i.e., Sigmoid, REL), also known as the non-linear mapping function, is a key module in CNNs. The fully connected layer maps the network features to the sample marker space, and the objective function is used to measure the error between the predicted value and the true sample marker. The cross-entropy loss function (i.e., SoftMax loss function) and the mean square error (i.e., L2 loss function) are the most used functions for classification and regression, respectively. Emotion classification in this context is a typical classification task, therefore, we take the cross-entropy loss function. In traditional CNN models, the input image is convolved several times to extract low-level features, with which, several high-level feature information is lost. As shown in Figure 1, we use two-layer convolutions, which can extract the low-level features while ensuring that the high-level feature information is obtained.

Inspired by VGG (23) and HFENet (24), the combination of two 3 × 3 convolutional kernels are used instead of unique 5 × 5 convolutional kernel, which improves the non-linear representation and reduces the network parameters. There is less information contained in the image over the same area, hence a convolutional kernel of size 7 × 7 is used in the first convolutional layer. The second convolutional layer uses a 5 × 5 convolutional kernel with the aim of extracting more detailed features.

The hand-drawn images contained colored drawings, which could correspond to different emotional statues. Hence, we use RGB three-channel convolution, and the input size of an image is 100 × 100 pixels. Convolutional layers are used for image feature extraction, and a pooling layer is adopted, meanwhile the second convolutional layer is used for image compression. The feature map of the previous layer is convolved with the convolution kernel of the current convolution layer. The convolution layer is defined as:


(1) $$\begin{array}{l} y^{j}=ReLu\left(b^{j}+\underset{j}{\sum }k^{ij*}x^{i}\right) \end{array}$$

where *x*^*i*^ denotes the *i*th input feature, *y*^*j*^ denotes the *j*th output feature, *k*^*ij*^ denotes the convolution kernel between *x*^*i*^ and *y*^*j*^, * denotes the convolution operation, the bias coefficient of the *j*th input feature is denoted by *b*^*j*^. The non-linear function *ReLu* is used as the activation function of convolution results, and the *ReLu* function is defined as:


(2) $$f(x)=\{\begin{array}{c} 0,\;x\leq 0\\ x,\;x>0 \end{array}$$

### The model settings

The initialization of CNN is done in two ways: (1) all-0 initialization and (2) random initialization. With reasonable data pre-processing and normalization, when the network converges to a stable state, the weights should remain half positive and half negative (when the expectation is 0). However, if all weights are zero, the output of different neurons in the network will be the same. The same output will lead to identical gradient updates, which will result in the updated parameters remaining in the same state, making it impossible to train the model. Random initialization avoids this problem by randomly setting the weights to a small random number close to 0.

The pooling layer is disabled to reduce feature loss. Each convolutional layer is followed by a Dropout layer, and the Dropout rate is set to 0.5. Such a setting not only reduces the number of parameters, but also reduces the training time of the network. The number of convolution kernels ranges from 16 to 32, with the final layer outputting 6, corresponding to the number of labeled categories in the classification dataset. The full parameters of the CNN structure are shown in Table 1.

**Table 1:** The full parameters of the adopted CNN structure.

**No**	**Layer**	**Operation**	**Type**	**Kernel size**	**Kernel amount**	**Output**
1	-	-	Input	-	-	100 × 100 × 3
2	L1	Convolution	Conv2D	7 × 7	16	94 × 94 × 16
3	-	Activation functions	ReLu	-	-	-
4	L2	Convolution	Conv2D	5 × 5	32	90 × 90 × 32
5	-	Activation functions	ReLu			-
6	L3	Fully connected layer	FC	-	-	256
7	-	Activation functions	ReLu	-	-	-
8	-	Dropout layer	Dropout	-	-	-
9	L4	Fully connected layer	FC	-	-	128
10	-	Activation functions	ReLu	-	-	-
11	L5	Fully connected layer	FC	-	-	6
13	L6	Activation functions	Sigmoid	-	-	6

The input of the model is designated to a 100 × 100 pixel image with three RGB channels. The input image will be convolved in the L1, which consists of 16 7 × 7 convolution kernels (step size is set to 1) and an activation function Relu. With the completion of convolution operations, the L1 outputs a set of 16-feature maps with a size of 94 × 94 pixels. Then, the image is convolved by L2, with 32 5 × 5 convolution kernels (a step size of 1, a padding of 0 and a Relu). Then, the L2 outputs a set of 32-feature maps with a size of 90 × 90 pixels. L3 is the first fully connected layer, and the activation function is Relu too. The Dropout layer outputs a 1 × 256 one-dimensional feature vector. L4 is the second fully connected layer, in which, the activation function is the Relu, and it's designed to output a 1 × 128 one-dimensional feature vector. L5 is the third fully connected layer, which outputs a 1 × 6 one-dimensional feature vector. L6 is a Sigmoid function, which outputs a 1 × 6 one-dimensional feature vector corresponding to the six categories of emotions.

### Components

In this paper, the multi-label classifier uses the Sigmoid function as the classifier. As illustrated in Figure 1, the nodes in the fully connected layer are connected to all nodes of the previous layer. It's designed to combine the features extracted from the previous side. The Sigmoid function is defined as below:


(3) $$\begin{array}{l} Sigmoid=\frac{1}{1+e^{-x}} \end{array}$$

Its range is in [0, 1]. The *Sigmoid* is also taken as the activation function in the output layer, meanwhile, the variance cost function is replaced by cross-entropy loss function to improve the training efficiency. The cross-entropy function is defined as below:


(4) $$\begin{array}{l} L\left(y,\;ŷ\right)=-\left[ŷ\log y+\left(1-ŷ\right)log\left(1-y\right)\right] \end{array}$$

where *y* and ŷ represent the predicted result and label. As argued in Lu et al. (24), the Nesterov-accelerated Adaptive Moment Estimation is adopted as the optimizer to calculate the adaptive learning rates for various parameters.

## Experiment and discussion

### Data collection and experimental procedure

**Figure 2:**
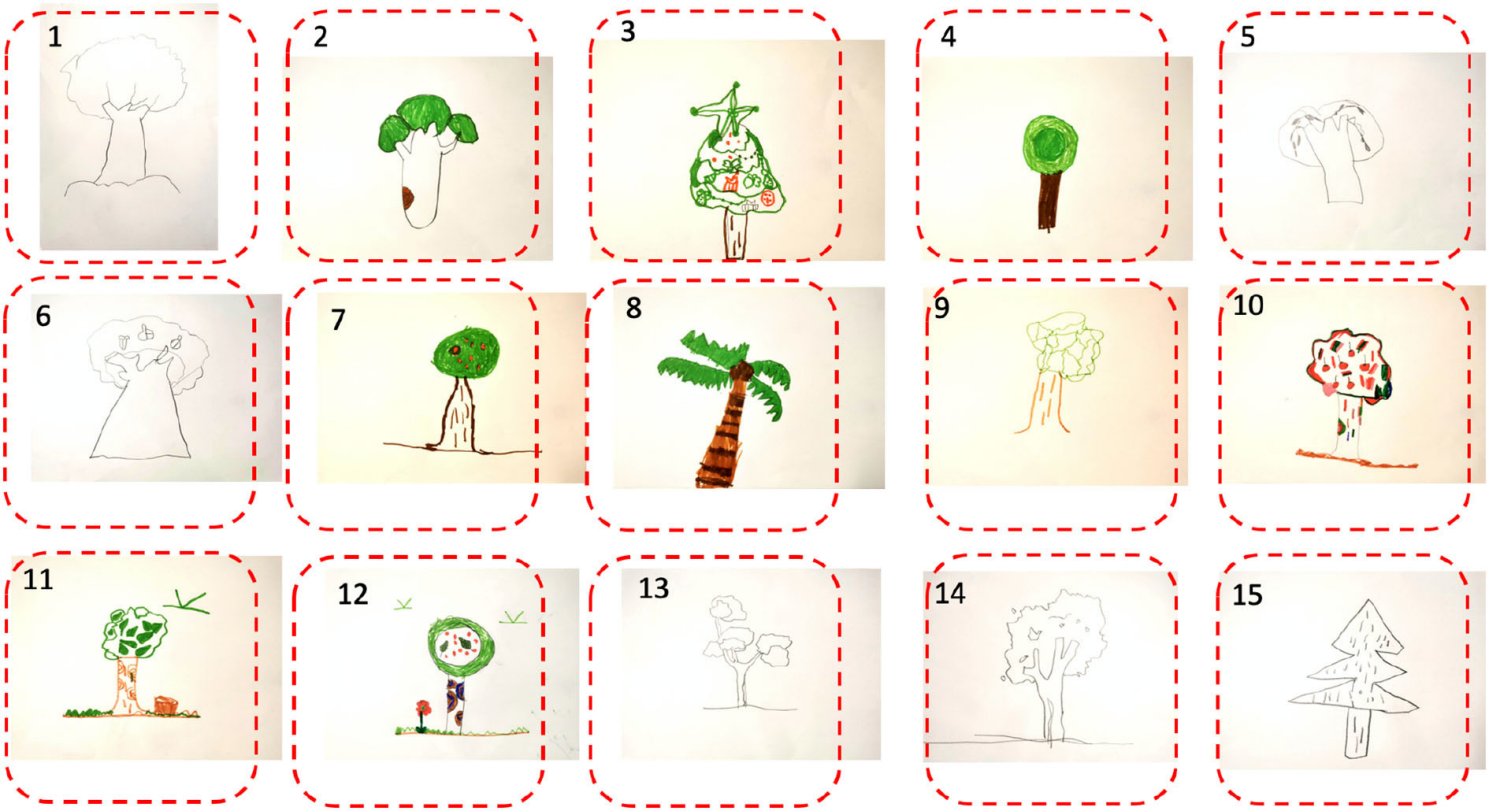
A group of hand-drawn paintings with the specified theme “tree.”

The experiment was carried out in a university at Hangzhou, China during 2019–2021. Two thousand one hundred three students were invited to join in the experiment (as shown in Table 2). Each participant was asked to read the experimental guide carefully and follow the instructions to complete the questionnaire (as shown in Table 2) and a drawing task on a A4 paper in a free style (as shown in Figure 2). The theme of the painting was designated to the tree, and all painting forms were welcome. The questionnaire form and the painting completed by a participant would be associated with his /her unique ID.

**Table 2:** The distribution of recruited participants in the experiment.

**Male**	**Female**	**Freshman**	**Sophomore**	**Junior**	**Senior**	
1,390	713	622	591	621	269	
**Year (18)**	**Year (19)**	**Year (20)**	**Year (21)**	**Year (22)**	**Year (23)**	**Year (24)**
132	310	535	222	613	189	102

Two thousand valid paintings were selected from the submitted paintings by these participants in this experiment. The size of the painting image input to the convolutional neural network was set to 100 × 100 pixels, hence the input image was deformed accordingly (e.g., we converted the rectangular image into a square image by filling in the blank around it).

Ekman's basic emotion theory has been proved and verified by several studies as the suitable basis for emotion analysis in learning environments. Hence, the emotional analysis by the psychologists is based on that theory, and the emotional statues reflected through the paintings focused on anxiety, stress, aggression, shyness, sense of security, engagement. In this experiment, we designed the questionnaire form (as shown in Table 3), and the participant could report his/her emotional statues in such a moment.

**Table 3:** The questionnaire designed for the participants to report their emotional statues.

**Please respond with an “x” to the following statements**.	**Yes (1)**	**No(0)**
Anxiety		
Stress		
Aggression		
Shy		
Sense of security		
Engagement		

A simple data conversion was also performed prior to the data labeling. As shown in Table 4, “1” denotes male, meanwhile “0” denotes female in the column “Gender.” In other columns, “1” denotes positive of the corresponding emotion, meanwhile, “0” denotes negative of the corresponding emotion. The answer of the questionnaire completed by the participant was converted and then used to label the corresponding painting. A professional psychologist was invited to review the completed questionnaire and the corresponding painting of the same participant, and then evaluate the answer of the participant. Amendment by the psychologist could be applied to the questionnaire form if necessary. That means the evaluation by the psychologist on the emotional status reflected by the painting is with higher prior than the reported emotional status by the participant.

**Table 4:** The data conversion of reported emotional statues by questionnaire form.

**No**.	**Gender**	**Happy**	**Surprise**	**Sadness**	**Anger**	**Fear**	**Disgust**
1	1	1	0	1	0	0	1
2	1	1	0	0	1	1	0
3	0	0	1	1	0	0	0
4	1	1	0	1	0	0	1
5	0	1	0	0	0	0	0
6	0	1	1	0	0	1	0
7	0	0	0	0	1	0	0
8	0	0	0	1	0	0	1
9	**1**	**1**	**1**	**0**	**0**	**0**	**0**
10	0	0	0	1	1	0	0

The bold values are the best values obtained in the experiment.

A total of 2,000 labeled paintings were obtained, and 1,600 of them were randomly selected as samples for model training. The rest of them (400) were used as test dataset. Meanwhile, the psychologist was also invited to make independent evaluation on the emotional statues reflected by these 400 paintings (test samples). The emotional statues of these 400 paintings reported by the psychologists would be compared with the results predicted by the trained model to obtain the accuracy of the CNN model.

### Evaluation metric

A classical metric for evaluating the classification accuracy was chosen. Given a test dataset for validation, prediction results can be divided to true positive (TP), true negative (TN), false positive (FP) and false negative (FN). Then the accuracy is formulized as below:


(5) $$\begin{array}{l} F\left(Acc\right)=\frac{numTP+numTN}{numTP+numTN+numFP+numFN} \end{array}$$

where *numTP* denotes the number of results actually true and predicted, *numTP* denotes the number of results not actually true but predicted, *numFP* denotes the number of result not actually true but predicted, *numFN* denotes the number of result actually true but not predicted, *numTN* denotes the number of results actually true and not predicted.

### Experimental setting

The server configuration was shown in Table 5. We used Python as the programming language to implement the algorithm used in this study and OpenCV to implement the image processing related functions. The experiment ran on a GPU with framework CUDA, and we used the framework Keras (https://keras.io) to implement deep learning related functions such as data pre-processing, network construction, data training, data prediction.

**Table 5:** Hardware and software settings for the experiment.

**Type**	**Setting**
CPU	Intel(R) Core(TM) i7-8700K
Memory	16 G
GPU	GeForce GTX 1080Ti
Operating system	Ubuntu 16.04
CUDA	CUDA 9.0
Programming language	Python 3.7
Deep learning framework	TensorFlow 1.2, Keras-gpu 2.2.4
Image processing library	OpenCV

### Experimental result

We adopted a cross validation based on the training dataset and validation dataset. The first convolutional layer (L1) consisted of a convolutional kernel in size of 3 × 3 and 16 groups of kernels, the second convolutional layer (L2) consisted of a convolutional kernel in size of 1 × 1 and 32 groups of kernels. The initial value of the Dropout rate was set to 0.5, the batch size was set to 40 and the training epoch was set to 100.

As shown in Table 6, the first convolutional layer consisted of 16 convolutional kernels and the second convolutional layer consisted of 32 convolutional kernels. Three rounds of training were conducted for each of these three convolution layers. It can be seen that the model with the first layer in size of 7 × 7 and the second layer in size of 5 × 5 achieved the highest accuracy separately.

**Table 6:** Comparison of classification accuracy of multiple binary classifiers.

**Kernel amount**	**Kernel size**	**Round of training**	**Happy**	**Surprise**	**Sadness**	**Anger**	**Fear**	**Disgust**	**Average Accuracy**
1,632	3 × 3 1 × 1	1	0.4191	0.5126	0.5704	0.8600	0.7513	0.7811	0.6491
		2	0.4512	0.5901	0.5991	0.7741	0.7632	0.4842	0.6102
		3	0.5101	0.5609	0.6004	0.8292	0.7339	0.7925	**0.6711**
	5 × 5 3 × 3	1	0.5298	0.5618	0.5698	0.7801	0.7189	0.6970	0.6429
		2	0.5312	0.6298	0.5710	0.8212	0.7331	0.7335	**0.6698**
		3	0.5201	0.5400	0.5192	0.8301	0.7231	0.5941	0.6210
	7 × 7 5 × 5	1	0.5231	0.5602	0.6154	0.8614	0.7616	0.7112	0.6721
		2	0.5491	0.5301	0.6716	0.8413	0.7531	0.7291	0.6790
		3	0.5613	0.5491	0.6271	0.8413	0.7531	0.8043	**0.6893**

The bold values are the best values obtained in the experiment.

Comparative experiments were also conducted as shown in Table 6, using multiple binary classifiers with the same size convolution kernel. As shown in Table 6, the accuracy of the method adopting the multi-label classifier was as high as 70%, while the multiple binary classifiers achieved a maximum accuracy of 68%.

As shown in Table 7, three independent experiments were performed in different parameter settings and the group with highest accuracy was recorded (marked in bold). As shown in Table 8, the highest accuracy was obtained when the first convolutional layer consisted of 32 kernels and the second convolutional layer consisted of 64 kernels.

**Table 7:** Comparison of experimental results with different kernel sizes.

**Kernel size**		**Trained accuracy**	**Tested accuracy**
**Layer 2**	**Layer 1**		
1 × 1	7 × 7	0.7216	0.6948
	5 × 5	0.7523	**0.7090**
	3 × 3	0.8669	0.6818
3 × 3	7 × 7	0.7207	0.6870
	5 × 5	0.7505	**0.6896**
	3 × 3	0.8594	0.6688
5 × 5	7 × 7	0.7216	**0.7210**
	5 × 5	0.7495	0.7013

The bold values are the best values obtained in the experiment.

**Table 8:** Comparison of experimental results with different numbers of kernel.

**Number of kernels**		**Trained accuracy**	**Tested accuracy**
***Filter 1***	***Filter 2***		
16	16	0.8780	0.6732
	32	0.8669	0.6753
	64	0.8594	**0.6832**
32	32	0.8650	0.6740
	64	0.8520	**0.6798**
	128	0.8501	0.6470

The bold values are the best values obtained in the experiment.

Tables 9, 10 demonstrate that a network with a Dropout rate of 0.5 and batch size of 40 worked best. The result of the tests showed that the correct test dataset for almost all parameters could reach over 67% and up to 70.3%. Even for a professional psychologist, the diagnostic process must be evaluated in combination with various kinds of factors such as the participant's specific performance, language and demeanor. The accuracy achieved by the machine learning method has therefore reached this and the standard of correct diagnostic rate for assisting psychologists. The best combination of parameters was determined as shown in Table 11.

**Table 9:** Comparison of experimental results with different dropout rates.

**Dropout rate**	**Trained accuracy**	**Tested accuracy**
0.2	0.8808	0.6710
0.3	0.8762	0.6732
0.4	0.8743	0.6753
0.5	0.7570	**0.6875**

The bold values are the best values obtained in the experiment.

**Table 10:** Comparison of experimental results with different batch sizes.

**Batch size**	**Trained accuracy**	**Tested accuracy**
20	0.8203	0.6688
30	0.8669	0.6623
40	0.8873	**0.6775**
50	0.9013	0.6658

The bold values are the best values obtained in the experiment.

**Table 11:** Optimal combination of parameters for CNN structure.

**Parameter type**	**Parameter setting**
Kernel size of layer 1	7 × 7
Kernel size of layer 2	5 × 5
Number of kernels of layer 1	32
Number of kernels of layer 2	64
Dropout	0.5
Batch	40
Tested accuracy	72.1%

Besides, traditional models such as LeNet and AlexNet were also applied to the experimental data. The features extracted by using HOG_SVM were simple and therefore could not be extracted for high-level features. The accuracy of LeNet in this experiment was 53.1%, which might be due to the absence of necessary features. The accuracy of AlexNet (69.1%) in the experiment was slightly inferior to the proposed method in this paper. Therefore, it could be determined that the proposed method combined with the Sigmoid classifier could classify the emotional statues of these paintings with multiple labels. The classification accuracy was 72.1%.

## Conclusion

In the learning environment, the learner's emotional status could bring significant impact on his/her concentration in the learning process and understanding of presentation. Capturing the learner's emotional statues could provide a reliable basis for the tutor to adjust pedagogical activities and strategies in a timely manner. We investigated existing studies regarding the emotion analysis based on the hand-drawn paintings, and in order to address the issues such as subjectivity and inefficiency in these existing studies, we proposed a CNN based method, and applied this method to carry out an experiment in an university in China, the result showed the proposed method was valid for emotional statues classification, and the model achieved classification accuracy of 72.1%.

Moreover, for CNN models, the increase in the training sample size will lead to an improvement in the performance of the model, but sample data collection and labeling is a costly task. In future, we will introduce methods such as horizontal flipping to expand the training dataset, and then further improve the classification accuracy.

## Data availability statement

The raw data supporting the conclusions of this article will be made available by the authors, without undue reservation.

## Author contributions

Research proposal: TH, CL, and CJ. Original draft preparation: JW, MW, and TH. Writing review and editing: MW, ZW, and CJ. All authors contributed to the article and approved the submitted version.

## Funding

This work is partially supported by the teaching reform project of Zhejiang University of Technology (No. GZ22241270052).

## Conflict of interest

The authors declare that the research was conducted in the absence of any commercial or financial relationships that could be construed as a potential conflict of interest.

## Publisher's note

All claims expressed in this article are solely those of the authors and do not necessarily represent those of their affiliated organizations, or those of the publisher, the editors and the reviewers. Any product that may be evaluated in this article, or claim that may be made by its manufacturer, is not guaranteed or endorsed by the publisher.
